# Association between Whole-Grain Intake and Obesity Defined by Different Anthropometric Indicators and Dose–Response Relationship Analysis among U.S. Adults: A Population-Based Study

**DOI:** 10.3390/nu16142373

**Published:** 2024-07-22

**Authors:** Yongjun Wang, Jing Feng, Tingting Liu, Zhaolong Gong, Qin Zhuo

**Affiliations:** Key Laboratory of Public Nutrition and Health of National Health Commission, National Institute for Nutrition and Health, Chinese Center for Disease Control and Prevention, Beijing 100050, China; wangyongjun519@163.com (Y.W.); fengjing0921@163.com (J.F.); liutt@ninh.chinacdc.cn (T.L.); gongzl@ninh.chinacdc.cn (Z.G.)

**Keywords:** whole grain, obesity, abdominal obesity, NHANES, dose–response relationship

## Abstract

Existing research shows an inconsistent correlation between whole-grain intake and obesity risk, with limited study on the dose–response relationship. Here, we aimed to examine this association and dose–response relationship among U.S. adults who participated in a NHANES (2003–2018). The intake of whole grain was collected and calculated from two rounds of 24 h dietary recall. Obesity was categorized based on body mass index (BMI) and waist circumference (WC). Weighted multivariable logistic regression models were used to calculate the odds of obesity according to whole-grain intake, and the dose–response relationship was modeled by restricted cubic spline regression. Among the 27,862 participants, 38.3% had general obesity, while 58.3% had abdominal obesity. After multivariate adjustment of potential confounders, the participants in the highest quintile of whole-grain intake had a lower prevalence of general obesity (OR 0.79; 95% CI 0.72–0.88) and abdominal obesity (OR 0.80; 95% CI 0.73–0.89) compared with those in the lowest category. Spline regression showed an inversely linear dose–response association between whole-grain intake and the prevalence of general obesity and abdominal obesity. In conclusion, a higher whole-grain intake was associated with lower odds of obesity, both general and abdominal. Our findings highlight the importance of increasing the whole-grain intake to prevent and manage obesity.

## 1. Introduction

Whole grains contain the bran, germ and endosperm of original grains. Therefore, compared with refined grains, whole grains are richer in dietary fiber, B vitamins, polyphenols, and minerals and are recognized as a healthy food [1,2]. Evidence from systematic reviews of RCTs and prospective studies indicated that increased whole-grain consumption was linked to reduced risks of diabetes, cardiovascular disease, colorectal and prostate tumors, and all-cause mortality [3,4,5,6]. A WHO guideline from 2023 recommends that the carbohydrate intake should come primarily from whole grains, vegetables, fruits, and pulses [7]. One of the key dietary principles of the Dietary Guidelines for Americans 2020–2025 recommends that at least half of the grain intake be whole grains [8].

The worldwide epidemic of obesity has attracted much attention, and with the continuous increase in the number of obese people, it has become a global public health problem. According to the World Obesity Map released by the World Obesity Federation in 2023, the prevalence of obesity (BMI ≥ 30 kg/m^2^) is expected to rise sharply from 14% to 24%, concerning about 2 billion people by 2035 [9]. Obesity is a multi-factorial disease due to genetic and environmental factors, unhealthy eating habits, lack of physical exercise, sedentary lifestyle [10,11]. Among several unhealthy dietary habits, insufficient whole-grain intake is a main factor for obesity. Whole grains have potential value in weight control as they are rich in dietary fiber, promote satiety, slow gastric emptying, and appetite control, and have a low energy density [12]. In addition, whole grains can regulate the gut microbiota and improve the body’s inflammatory state, which is beneficial to weight control [13,14]. Epidemiological investigations and clinical trials have explored the relationship between the consumption of whole grains and obesity. Some studies have shown that increased consumption of whole grains can lead to weight loss or decrease the percentage of body fat [15]. A meta-analysis in 2019 including 11 studies and 498 participants revealed an association between higher whole-grain intake and larger body weight change [3]. However, a meta-analysis of RCTs including 26 studies and 2060 participants found that whole grains did not play a role in weight control [16]. The existing research evidence shows an inconsistent correlation between whole-grain intake and the risk of obesity. In addition, the dose–response relationship between whole-grain intake and obesity has not been fully explored.

In the current study, our primary objective was to scrutinize the correlations between whole-grain intake and obesity defined by both BMI and waist circumference among participants in a representative National Health and Nutrition Examination Survey (NHANES) of U.S. adults. Furthermore, we intended to delve deeper into the dose–response relationship between whole-grain intake and the likelihood of obesity.

## 2. Materials and Methods

### 2.1. Study Population

In this study, we used 8 cycles of datasets from a NHANES conducted from 2003–2004 to 2017–2018. The NHANES comprises a series of surveys designed to assess the health and nutritional status of the civilian population in the United States using a nationally representative sample. The survey used a complex, stratified, multi-staged probability cluster sampling design to select the non-institutionalized civilian population in the United States [17]. The investigators conducted the questionnaires at their own homes and a series of tests at the mobile examination center to collect demographic, socio-economic, diet, and health-related data. Specific methods and more details of the survey can be found on the website www.cdc.gov/nchs/nhanes (accessed on 20 June 2024). The NHANES was approved by the National Center for Health Statistics (NCHS) ethics review board, and all participants provided written informed consent.

**Figure A1:**
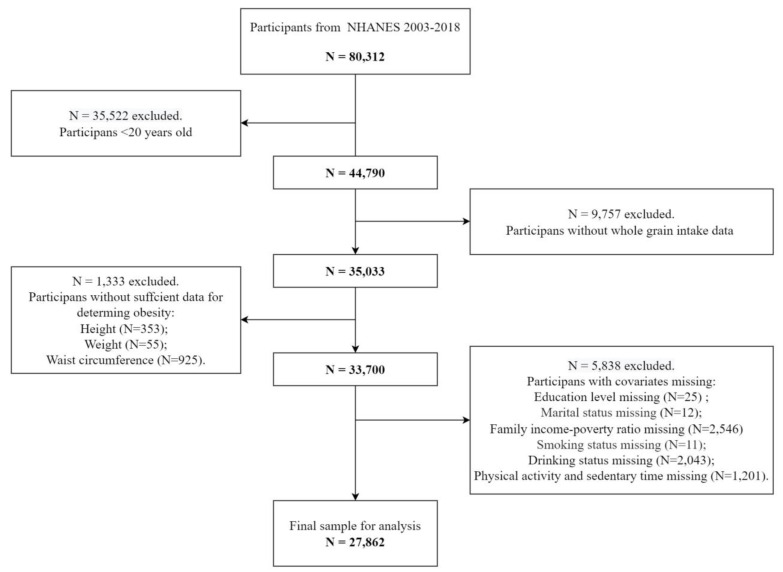
Flowchart of the study population in the NHANES from 2003 to 2018.

Of the 80,312 people who participated in the NHANES 2003–2018 survey, 44,790 participants were 20 years or older, of whom 35,033 completed a two-day dietary review to provide whole-grain intake data. We excluded those whose height, weight, and waist circumference were not known (*n* = 1333) and those for whom information about key covariates was lacking (*n* = 5838). Finally, 27,862 participants were included in the current study (Figure A1).

### 2.2. Assessment of Individual Whole-Grain Intake

Dietary intake data were collected through two rounds of 24 h recall interviews conducted by trained interviewers. The first interview was in person at the Mobile Examination Center (MEC), and the second was via phone within 3–10 days, avoiding the same weekday. The dietary intake of the participants was averaged from both interviews. Whole-grain foods were defined by applying the definition of the Dietary Guidelines for Americans (DGA) that define whole-grain food as food in which ≥50% of the total grain weight is represented by whole-grain ingredients [8]. We identified whole-grain foods in the Food and Nutrition Database for Dietary Studies (FNDDS) for each survey cycle and estimated their consumption. Using the Food Patterns Equivalents Database (FPED) and My Pyramid Equivalents Database (MPED), we quantified whole grain, refined grain, and total grain per 100 g of each food [18]. Finally, the daily whole-grain, refined grain, and total grain consumption was calculated for each participant. Furthermore, the percentage of whole grains in the total grains consumed was calculated.

### 2.3. Body Measures and Assessment of Obesity

All participants aged 20 years and above underwent standardized anthropometric measurements of weight, height, and waist circumference, conducted by trained personnel [19]. The body mass index (BMI) was computed by dividing the weight in kilograms by the square of the height in meters. Obesity was categorized based on BMI criteria, with a threshold of BMI ≥ 30 indicating generalized obesity [20]. Additionally, abdominal obesity was identified using waist circumference measurements, with cut-off values of ≥102 cm for males and ≥88 cm for females [21].

### 2.4. Assessment of Covariates

In face-to-face interviews, standardized questionnaires were used to collect information on age, gender, race or ethnicity, education, marital status, family income, smoking status, drinking status, physical activity, and sitting time. Races were divided into Non-Hispanic Whites, Non-Hispanic Blacks, Mexican Americans, and other races. The education level was divided into below high school, high school, and college or above. The marital status was classified as married/cohabitation, never married, and widowed/divorced/separated. The poverty/income ratio (PIR) was divided into three grades (<1.0, 1.0–3.0, and >3.0). The smoking status was classified as never, former, or current smoker. The drinking status was classified as heavy drinker, mild to moderate drinker, former drinker, and non-drinker. The physical activity level was classified as inactive, insufficiently active, and active. According to the sitting time, sedentary time was ranked as ≥6 h, from 4 to <6 h, and <4 h. Dietary information was collected from two 24 h dietary reviews, and the total intakes of energy, fruit, vegetable, milk, meat, and added sugar were obtained based on the FNDDS and FPED.

### 2.5. Statistical Analysis

According to the NHANES data analysis guidelines, in order to ensure the national representation of the analysis, we considered the complex survey design factors of the NHANES, including sample weight, clustering, and stratification. We used the mean ± SE for continuous variables and *n* (%) for classified variables when describing the characteristics of the study participants. A weighted chi-square test and weighted linear regression model were used to test the differences between different groups of whole grains. Weighted multivariate adjusted logistic regression was used to test the independence between whole-grain intake and obesity, adjusting for confounding variables. Weighted multivariate adjusted logistic regression models were used to estimate the ORs and 95% CIs of obesity according to tertiles of whole-grain intake. OR can be calculated using the following formula $OR=\exp\left(\beta \right),\;where\;\beta$ is the coefficient of logistic regression. Based on previous literature and biological rationality, we constructed three statistical models. Model 1 adjusted for age, sex, and race. Model 2 further adjusted for education level, marital status, PIR, smoking status, drinking status, physical activity, sedentary time. Model 3 further adjusted for energy intake, vegetable intake, fruit intake, meat intake, dairy intake, and added sugar intake. Linear trend testing was achieved by assigning a median to each category as a continuous variable. In addition, a stratified analysis was conducted, and the *p* value of the product of whole-grain intake and stratified variables was used to test the interaction. Finally, the restricted cubic spline regression model of 3 knots (5th, 50th, and 95th percentile) was used to explore the dose–response relationship between whole-grain intake and its percentage and obesity for the total number of participants and for male and female participants, adjusting the same variables as in model 3. Nonlinearity was evaluated using likelihood ratio tests, comparing models incorporating the cubic spline term with those excluding it.

All statistical analyses were performed using R (http://www.R-project.org, version 4.3.1) (accessed on 20 June 2024). Statistical significance was defined as a two-sided *p*-value < 0.05.

## 3. Results

### 3.1. Description of the Characteristics of the Participants

The basic characteristics of the 27,862 participants are summarized according to the tertile level of whole-grain intake in Table 1. The weighted mean age of the study participants was 47.1 years, and 48.3% were male. The participants with higher whole-grain intake were more likely to be female, Non-Hispanic White, married, have a higher income, have never smoked, drink alcohol moderately or lightly, and present a higher physical activity level and a longer sitting time. Meanwhile, they tended to eat more vegetables, fruits, and dairy products and less meat and added sugar. Among the 27,862 participants, a total of 10,661 (38.3%) participants were identified as presenting general obesity, and 16,243 (58.3%) participants were identified as presenting abdominal obesity. Whether obesity was defined by BMI or waist circumference, the high whole-grain intake group had a lower obesity rate.

### 3.2. Description of Multivariable Associations between Whole-Grain Intake and Obesity

After multivariate adjustments of potential confounders, all three models demonstrated an inverse relationship between whole-grain intake and the prevalence of obesity (Table 2). In the fully adjusted model, the ORs (95% CIs) across the tertiles of whole-grain intake were 1.00, 0.87 (0.79, 0.96), and 0.79 (0.72, 0.88) for the prevalence of general obesity defined by BMI (*p* for trend = 0.011) and 1.00, 0.89 (0.81,0.98), and 0.80 (0.73,0.89) for the prevalence of abdominal obesity defined by waist circumference (*p* for the trend = 0.034). After a similar multivariable adjustment, the ORs (95% CIs) were 0.91 (0.87, 0.96) and 0.90 (0.86, 0.94) for a per-SD change in whole-grain intake, respectively.

### 3.3. Dose–Response Associations between Whole-Grain Intake or Proportion of Whole-Grain Intake and Obesity

#### 3.3.1. Dose–Response Associations between Whole-Grain Intake and Obesity

**Figure 1:**
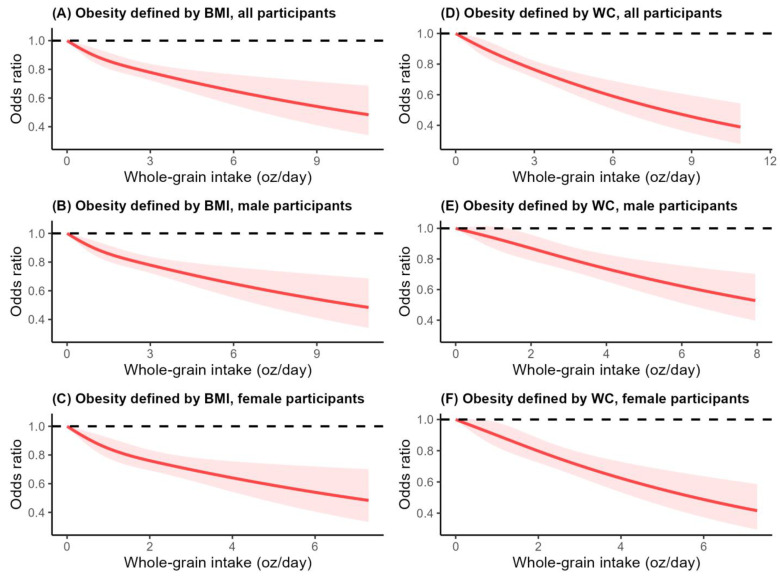
Multivariable adjusted, dose–response associations between whole-grain intake and obesity for adults who participated in the NHANES (2003–2018). Association between whole -grain intake and obesity defined by body mass index for (**A**) all participants, (**B**) male participants, (**C**) female participants. Associations between whole-grain intake and obesity defined by waist circumference for (**D**) all participants, (**E**) male participants, (**F**) female participants. A restricted cubic spline regression model with three knots (5th, 50th, and 95th percentiles) was employed to estimate the dose–response associations between whole-grain intake and obesity. Odds ratios were adjusted for age, sex, race, education level, marital status, PIR, smoking status, drinking status, physical activity, sedentary time, energy intake, vegetable intake, fruit intake, meat intake, dairy intake, and added sugar intake. Light red shaded areas represent the 95% CI of odds ratios. *p* > 0.05 for non-linearity in all cases.

After multivariate adjustments, inversely linear dose–response associations between whole-grain intake and obesity were observed for all NHANES (2003–2018) participants (Figure 1A), as well as for the male participants (Figure 1B) and the female participants (Figure 1C) when general obesity was considered, defined by BMI (*p* non-linearity >0.05). Meanwhile, similar linear dose–response associations were also observed when abdominal obesity was considered, defined by waist circumference (Figure 1D–F) (*p* non-linearity > 0.05).

#### 3.3.2. Dose–Response Associations between Proportion of Whole-Grain Intake and Obesity

**Figure 2:**
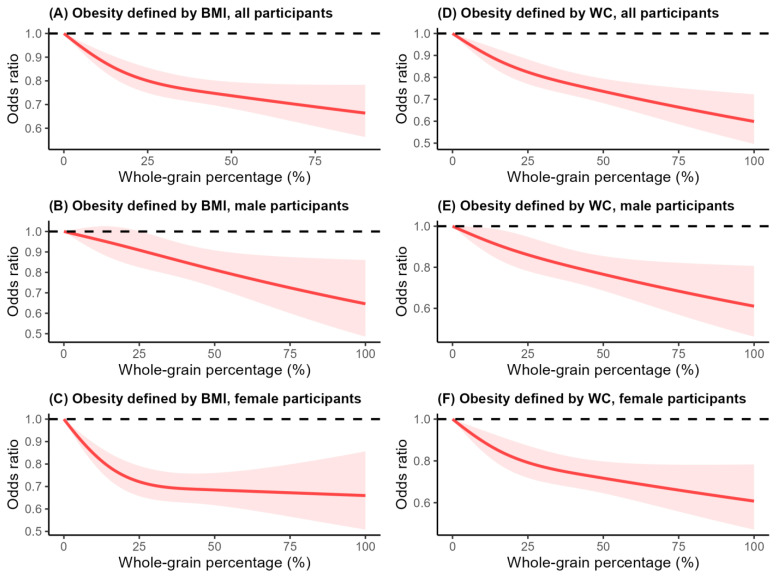
Multivariable adjusted, dose–response associations between whole-grain intake percentage of total grain intake and obesity for adults who participated in the NHANES (2003–2018). Association between whole-grain intake percentage and obesity defined by body mass index for (**A**) all participants, (**B**) male participants, (**C**) female participants. Associations between whole-grain intake percentage and obesity defined by waist circumference among (**D**) all participants, (**E**) male participants, (**F**) female participants. A restricted cubic spline regression model with three knots (5th, 50th, and 95th percentiles) was employed to estimate the dose–response associations between whole-grain intake percentage and obesity. Odds ratios were adjusted for age, sex, race, education level, marital status, PIR, smoking status, drinking status, physical activity, sedentary time, energy intake, vegetable intake, fruit intake, meat intake, dairy intake, and added sugar intake. Light red shaded areas represent the 95% CI of odds ratios. *p* < 0.01 for non-linearity for (**A**) and (**C**), *p* > 0.05 for non-linearity in the other cases.

Furthermore, we explored the dose–response relationship between the percentage of whole-grain intake and the incidence of obesity. There was a linear dose–response relationship between the percentage of whole-grain intake and obesity defined by waist circumference (Figure 2D–F) (*p* non-linearity > 0.05). An inversely linear correlation between the proportion of whole-grain intake and obesity was also observed for the male participants (Figure 2B) (*p*-linearity > 0.05). However, a non-linear relationship between the proportion of whole-grain intake and obesity defined by BMI for the total population and the female participants (both *p*-non-linearity < 0.01) was observed, and the critical values were 24.0% and 44.2%, respectively (Figure 2A,C).

### 3.4. Stratified Analyses of the Associations between Whole-Grain Intake and Obesity

The associations of whole-grain intake with obesity in stratified analyses are shown in Table 3. In the subgroup analysis, there was a statistical correlation between whole-grain intake and obesity defined by BMI for the Non-Hispanic White participants, with OR of 0.75 (95% CI 0.66, 0.87), but not for the Non-White participants. A statistical correlation between whole-grain intake and obesity defined by BMI was found for the married/cohabiting and never-married participants, but not for the widowed/divorced/separated participants. There was a statistical correlation between whole-grain intake and obesity defined by BMI for non-smokers and former smokers, but not for current smokers. A correlation between whole-grain intake and obesity (defined by BMI) was observed in relation to gender and smoking status (the *p* values for the interactions were 0.03 and 0.03, respectively). Similar results were observed in the stratified analysis of the association between whole-grain intake and abdominal obesity (defined by waist circumference).

## 4. Discussion

In this study, we used a nationally representative sample of U.S. adults to explore the relationship between whole-grain intake and obesity defined by different anthropometric indicators. We found that a higher whole-grain intake was associated with a lower incidence of general obesity and abdominal obesity. Additionally, there were linear dose–response associations between whole-grain intake and general obesity and abdominal obesity. These findings strengthen the previously established link between whole-grain intake and obesity and provide new ideas for preventing and controlling obesity by increasing whole-grain intake.

An inverse correlation between high whole-grain intake and obesity was found in previous studies. Pamela L. et al. found that whole-grain intake was inversely associated with obesity defined by BMI based on data from the Multi-Ethnic Study of Atherosclerosis [22]. In a large area-based population, Mostad et al. found that central obesity was associated with decreased consumption of whole grains [23]. A systematic review of fifteen observational studies including 119,829 male and female participants revealed that the consumption of three daily servings of whole grains was associated with lower BMI as well as central adiposity [24]. These results are consistent with our findings. A number of systematic analyses also summarized the relationship between whole-grain intake and body weight based on meta-analyses of prospective studies and RCTs. The results of the meta-analysis of prospective studies are consistent with our study results [3,25,26]; however, the meta-analysis results of RCTs showed that increasing the whole-grain intake had no effect on body weight control [27,28]. However, most of the current whole grain-related RCTs have research limits such as insufficient time for the whole-grain interventions and low intervention doses. Moreover, some RCTs were based on a single type of whole grains, but different types of whole grains have different effects. There may be large differences in the beneficial components of whole grains, which may not fully reflect the comprehensive effects of whole grains.

To analyze the relationship between whole-grain intake and obesity, we also used spline regression. We found that for both general obesity and central obesity, as the whole-grain intake increased, the obesity rate decreased, and the relationship had a linear trend. The dose–response relationships between whole-grain intake and several non-communicable diseases and their risk factors were explored in recent studies. Andrew et al. found that a high intake of whole grains was beneficial for several health outcomes in a linear manner [3], while Dagfinn et al. found a non-linear relationship [29]. Although evidence from RCT studies indicated that a high intake of whole grains was beneficial for reducing weight gain [30], due to the inconsistent definition of whole grains used in various studies [31], resulting in a relatively large heterogeneity between studies, the dose–response relationship between whole-grain intake and body weight cannot be established. Our study attempted to explore the dose–response relationship between whole-grain consumption and obesity and found a linear relationship. This provides some evidence for increased health benefits from increasing the whole-grain intake. In addition to the whole-grain intake itself, we also tried to further explore the relationship between the proportion of whole grains in the total grain intake and obesity. Interestingly, we found that the proportion of whole grains in the total cereal intake was linearly related to central obesity, but there was a nonlinear relationship with general obesity. When the proportion of whole grains reached a certain level (24.0% for the total number of participants, 44.2% for the female participants), the effect of whole grains on weight control was no longer significant. This result suggests that maintaining an appropriate ratio between the amount of whole grains and that of refined grains is more meaningful for obesity, especially for general obesity. Of course, the relationship may be worthy of further exploration.

Our results suggest that a higher whole-grain intake was associated with lower odds of both general obesity and central obesity. A high consumption of whole grains was found to be beneficial in weight control, and the underlying mechanisms can be attributed to several factors. Firstly, compared to refined grains, whole grains provide a lower caloric supply [32]. Secondly, whole grains are rich in dietary fiber, which enhances the sensation of satiety and slows down gastric emptying [33], thereby curbing appetite and reducing food intake. Furthermore, the fermentation of whole grains in the intestine generates significant amounts of short-chain fatty acids, which not only modulate the intestinal microbiota but also maintain blood sugar homeostasis and improve appetite regulation [34,35,36].

The highlights of this study are the following. Firstly, this study was based on a nationally representative large sample of U.S. adults. Secondly, when studying the relationship between whole-grain intake and obesity, we considered general obesity defined by BMI and central obesity defined by waist circumference. In addition, when exploring the relationship between whole-grain intake and obesity, the dose–response relationship was analyzed. Furthermore, the appropriate percentage of whole grains for obese people was also explored by using spline regression.

Of course, this study also has some potential limitations worth considering. Firstly, this is a cross-sectional study, and causal associations cannot be drawn due to its limitations. Therefore, follow-up large-scale standard RCT studies need to be designed to further explore the relationship between whole grains and obesity. Secondly, our findings cannot be extrapolated to other populations, since the study was conducted only among U.S. adults. In the future, data from multiple regions will need to be combined. Third, whole-grain intake information was obtained from self-reported dietary recalls, which inevitably have a certain recall bias.

## 5. Conclusions

In conclusion, a higher whole-grain intake was associated with lower odds of obesity, in terms of both general and abdominal obesity. Furthermore, whole-grain intake correlated linearly with the prevalence of general and abdominal obesity. Our findings highlight the potential benefits of whole grains in weight control and provide further support for recommendations of increasing the whole-grain intake to maintain a healthy weight in obese people. Rigorously well-designed clinical intervention trials are needed to strengthen the evidence for the association between whole-grain consumption and obesity.

## Figures and Tables

**Table 1:** The characteristics of the participants according to their whole-grain intake.

Characteristic	Whole-Grain Intake (oz/day)				*p*-Value
	Total	Tertial 1 (<0.08)	Tertial 2 (0.08–0.91)	Tertial 3 (>0.91)	
No. of participants	27,862	9273	9273	9316	
Age, y	47.1 ± 0.2	44.1 ± 0.3	47.2 ± 0.3	49.6 ± 0.4	<0.001
Sex, *n* (%)					<0.001
Female	14,403 (51.7)	4422 (48.2)	5350 (57.1)	4631 (50.3)	
Male	13,459 (48.3)	4851 (51.8)	3923 (42.9)	4685 (49.7)	
Race, *n* (%)					<0.001
Non-Hispanic White	13,014 (46.7)	3672 (61.6)	4368 (70.7)	4974 (75.8)	
Non-Hispanic Black	5738 (20.6)	2157 (13.6)	1979 (10.7)	1602 (7.8)	
Mexican American	4269 (15.3)	1734 (10.9)	1461 (8.1)	1074 (5.5)	
Other race	4841 (17.4)	1710 (13.9)	1465 (10.5)	1666 (10.9)	
Education level, *n* (%)					<0.001
Below high school	6224 (22.3)	2749 (20.2)	1962 (13.1)	1513 (9.7)	
High school	6434 (23.1)	2359 (27.4)	2162 (23.6)	1913 (18.8)	
College or above	15,204 (54.6)	4165 (52.4)	5149 (63.3)	5890 (71.5)	
Marital status, *n* (%)					<0.001
Married/cohabiting	17,102 (61.4)	5536 (61.7)	5686 (64.6)	5880 (65.9)	
Never married	4785 (17.2)	1872 (21.1)	1563 (17.8)	1350 (15.6)	
Widowed/divorced/separated	5975 (21.4)	1865 (17.3)	2024 (17.6)	2086 (18.5)	
PIR, *n* (%)					<0.001
<1.0	5328 (19.1)	2325 (18.1)	1686 (12.2)	1317 (9.3)	
1.0–3.0	11,706 (42)	4121 (39.3)	3858 (35.7)	3727 (32.7)	
>3.0	10,828 (38.9)	2827 (42.6)	3729 (52.1)	4272 (58.0)	
Height, cm	168.8 ± 0.1	168.8 ± 0.2	168.1 ± 0.1	169.5 ± 0.2	<0.001
Weight, kg	82.7 ± 0.2	84.1 ± 0.4	82.1 ± 0.3	82.0 ± 0.4	<0.001
BMI, kg/m^2^	28.9 ± 0.1	29.5 ± 0.1	29.0 ± 0.1	28.4 ± 0.1	<0.001
Waist circumference, cm	99.1 ± 0.2	100.1 ± 0.3	98.9 ± 0.3	98.3 ± 0.3	<0.001
Smoking status, *n* (%)					<0.001
Never	15,239 (54.7)	4668 (50.3)	5154 (56.7)	5417 (58.3)	
Former	7075 (25.4)	2072 (21.8)	2344 (24.2)	2659 (28.5)	
Now	5548 (19.9)	2533 (27.9)	1775 (19.1)	1240 (13.1)	
Drinking status, *n* (%)					<0.001
Never	3751 (13.5)	1163 (10.5)	1294 (10.4)	1294 (11.3)	
Former	4800 (17.2)	1596 (14.1)	1583 (13.3)	1621 (13.9)	
Mild to moderate	13,927 (50)	4139 (47.8)	4704 (55.8)	5084 (59.6)	
Heavy	5384 (19.3)	2375 (27.6)	1692 (20.5)	1317 (15.3)	
Physical activity, *n* (%)					<0.001
Inactive	7193 (25.8)	2699 (24.7)	2411 (20.3)	2083 (17.9)	
Insufficiently active	5640 (20.2)	1831 (21.1)	1993 (21.9)	1816 (19.2)	
Active	15,029 (53.9)	4743 (54.2)	4869 (57.8)	5417 (62.9)	
Sedentary time, *n* (%)					0.02
≥6	15,765 (56.6)	4993 (58.1)	5337 (58.5)	5435 (60.7)	
4 to <6	6110 (21.9)	2082 (21.9)	1967 (22.7)	2061 (22.2)	
0 to <4	5987 (21.5)	2198 (20.0)	1969 (18.8)	1820 (17.1)	
Vegetable intake, cup/day	1.6 ± 0.0	1.5 ± 0.0	1.6 ± 0.0	1.8 ± 0.0	<0.001
Fruit intake, cup/day	1.0 ± 0.0	0.7 ± 0.0	0.9 ± 0.0	1.3 ± 0.0	<0.001
Meat intake, oz/day	4.9 ± 0.0	5.1 ± 0.1	4.8 ± 0.1	4.7 ± 0.1	<0.001
Dairy intake, cup/day	1.6 ± 0.0	1.4 ± 0.0	1.6 ± 0.0	1.8 ± 0.0	<0.001
Added sugar intake, tsp/day	17.2 ± 0.2	18.9 ± 0.3	17.2 ± 0.3	15.6 ± 0.2	<0.001
Energy intake, kcal/day	2063.3 ± 10.3	1992.4 ± 16.0	2002.3 ± 14.6	2183.6 ± 14.4	<0.001
Obesity defined by Body mass index, *n* (%)					<0.001
Non-obesity	17,201 (61.7)	5516 (59.4)	5626 (63.2)	6059 (66.4)	
Obesity	10,661 (38.3)	3757 (40.6)	3647 (36.8)	3257 (33.6)	
Obesity defined by Waist circumference, *n* (%)					0.02
Non-obesity	11,619 (41.7)	3911 (42.4)	3673 (42.2)	4035 (45.0)	
Obesity	16,243 (58.3)	5362 (57.6)	5600 (57.8)	5281 (55.0)	

Continuous variables are presented as means ± SEs. Categorical variables are presented as *n* (%). PIR, poverty/income ratio. BMI, body mass index.

**Table 2:** Association between whole-grain intake and obesity defined by body mass index or waist circumference.

Characteristic	Whole-Grain Intake			*p* for Trend	Per-SD Increase
	Tertile 1	Tertile 2	Tertile 3		
General obesity defined by body mass index					
No. obesity/total	3757/9273	3647/9273	3257/9316		
Model 1	1.00	0.84 (0.77, 0.93)	0.74 (0.68, 0.82)	0.001	0.89 (0.85, 0.93)
Model 2	1.00	0.86 (0.78, 0.95)	0.77 (0.69, 0.85)	0.007	0.90 (0.86, 0.94)
Model 3	1.00	0.87 (0.79, 0.96)	0.79 (0.72, 0.88)	0.011	0.91 (0.87, 0.96)
Abdominal obesity defined by waist circumference					
No. obesity/total	3911/9273	3673/9273	4035/9316		
Model 1	1.00	0.86 (0.78, 0.94)	0.75 (0.68, 0.83)	0.003	0.88 (0.84, 0.92)
Model 2	1.00	0.88 (0.80, 0.97)	0.78 (0.71, 0.86)	0.024	0.89 (0.85, 0.93)
Model 3	1.00	0.89 (0.81, 0.98)	0.80 (0.73, 0.89)	0.034	0.90 (0.86, 0.94)

Model 1 was adjusted for age, sex, and race. Model 2 was further adjusted for education level, marital status, PIR, smoking status, drinking status, physical activity, sedentary time. Model 3 was further adjusted for energy intake, vegetable intake, fruit intake, meat intake, dairy intake, and added sugar intake.

**Table 3:** Stratified analyses of the associations (ORs, 95% CIs) between whole-grain intake and obesity among adults in the NHANES (2003–2018).

Characteristic	Whole-Grain Intake			*p* for Trend	*p* for Interaction
	T1	T2	T3		
General obesity defined by body mass index					
Age					0.79
≤60	1.00	0.90 (0.81, 1.01)	0.82 (0.74, 0.92)	<0.001	
>60	1.00	0.81 (0.66, 1.00)	0.75 (0.62, 0.92)	0.38	
Sex					0.03
Female	1.00	0.85 (0.74, 0.96)	0.73 (0.63, 0.85)	<0.001	
Male	1.00	0.90 (0.78, 1.04)	0.86 (0.75, 0.99)	0.15	
Race					0.25
Non-Hispanic White	1.00	0.85 (0.74, 0.96)	0.75 (0.66, 0.87)	0.03	
Non-Hispanic Black	1.00	0.97 (0.83, 1.15)	0.92 (0.78, 1.09)	0.64	
Mexican American	1.00	0.85 (0.65, 1.12)	0.91 (0.72, 1.16)	0.41	
Other race	1.00	0.93 (0.77, 1.12)	0.86 (0.69, 1.06)	0.50	
Marital status					0.49
Married/cohabiting	1.00	0.89 (0.78, 1.02)	0.75 (0.67, 0.85)	0.16	
Never married	1.00	0.83 (0.67, 1.03)	0.80 (0.65, 0.99)	0.88	
Widowed/divorced/separated	1.00	0.89 (0.73, 1.08)	0.94 (0.76, 1.17)	0.23	
Smoking status					0.03
Never	1.00	0.81 (0.71, 0.92)	0.71 (0.62, 0.81)	<0.001	
Former	1.00	0.97 (0.79, 1.19)	0.79 (0.66, 0.96)	0.01	
Now	1.00	0.90 (0.74, 1.09)	1.09 (0.88, 1.36)	0.60	
Drinking status					0.70
Never	1.00	0.80 (0.62, 1.02)	0.76 (0.58, 1.00)	0.09	
Former	1.00	0.87 (0.68, 1.11)	0.73 (0.57, 0.93)	0.29	
Mild to moderate	1.00	0.86 (0.74, 0.98)	0.77 (0.67, 0.88)	0.07	
Heavy	1.00	0.95 (0.76, 1.19)	0.93 (0.73, 1.19)	0.56	
Physical activity					0.08
Inactive	1.00	0.83 (0.67, 1.02)	0.86 (0.69, 1.06)	0.08	
Insufficiently active	1.00	0.81 (0.66, 0.98)	0.85 (0.69, 1.05)	0.04	
Active	1.00	0.92 (0.81, 1.05)	0.76 (0.66, 0.87)	0.01	
Sedentary time					0.47
≥6	1.00	0.82 (0.72, 0.93)	0.79 (0.69, 0.91)	0.003	
4 to <6	1.00	0.97 (0.77, 1.22)	0.82 (0.65, 1.04)	0.09	
0 to <4	1.00	0.96 (0.76, 1.20)	0.75 (0.60, 0.94)	0.02	
Abdominal obesity defined by waist circumference					
Age					0.90
≤60	1.00	0.92 (0.82, 1.04)	0.86 (0.76, 0.96)	0.01	
>60	1.00	0.85 (0.70, 1.04)	0.82 (0.67, 1.01)	0.68	
Sex					0.07
Female	1.00	0.85 (0.74, 0.96)	0.73 (0.63, 0.85)	<0.001	
Male	1.00	0.90 (0.78, 1.04)	0.86 (0.75, 0.99)	0.15	
Race					0.08
Non-Hispanic White	1.00	0.86 (0.75, 0.98)	0.76 (0.67, 0.87)	0.06	
Non-Hispanic Black	1.00	0.95 (0.81, 1.13)	0.92 (0.75, 1.12)	0.80	
Mexican American	1.00	0.79 (0.61, 1.03)	0.89 (0.71, 1.13)	0.25	
Other race	1.00	1.09 (0.87, 1.38)	0.98 (0.78, 1.23)	0.31	
Marital status					0.89
Married/cohabiting	1.00	0.87 (0.76, 0.99)	0.76 (0.67, 0.87)	0.07	
Never married	1.00	0.95 (0.77, 1.18)	0.84 (0.67, 1.05)	0.38	
Widowed/divorced/separated	1.00	0.94 (0.77, 1.16)	0.94 (0.72, 1.22)	0.58	
Smoking status					0.01
Never	1.00	0.82 (0.72, 0.94)	0.72 (0.63, 0.83)	<0.001	
Former	1.00	0.96 (0.78, 1.19)	0.79 (0.64, 0.97)	0.02	
Now	1.00	0.94 (0.78, 1.13)	1.13 (0.89, 1.43)	0.39	
Drinking status					0.28
Never	1.00	0.85 (0.65, 1.12)	0.80 (0.60, 1.07)	0.26	
Former	1.00	0.80 (0.63, 1.01)	0.68 (0.53, 0.88)	0.07	
Mild to moderate	1.00	0.90 (0.78, 1.04)	0.77 (0.67, 0.88)	0.28	
Heavy	1.00	0.93 (0.73, 1.18)	1.01 (0.79, 1.30)	0.99	
Physical activity					0.08
Inactive	1.00	0.78 (0.64, 0.95)	0.85 (0.69, 1.05)	0.01	
Insufficiently active	1.00	0.85 (0.68, 1.07)	0.86 (0.66, 1.12)	0.17	
Active	1.00	0.94 (0.84, 1.06)	0.78 (0.68, 0.89)	0.01	
Sedentary time					0.16
≥6	1.00	0.80 (0.70, 0.92)	0.73 (0.64, 0.84)	0.003	
4 to <6	1.00	1.08 (0.87, 1.33)	0.89 (0.73, 1.10)	0.25	
0 to <4	1.00	0.97 (0.78, 1.20)	0.94 (0.76, 1.16)	0.54	

## Data Availability

Data can be obtained from the NHANES database (https://www.cdc.gov/nchs/nhanes/) (accessed on 20 June 2024).
